# The influence of face mask color on perceptions of African American and white men

**DOI:** 10.1007/s10339-024-01196-y

**Published:** 2024-05-10

**Authors:** Lauren A. Morris, Doris G. Bazzini, Christopher J. Holden, Savannah J. Lee

**Affiliations:** Department of Psychology, Appalachian State University, 222 Joyce Lawrence Lane, Boone, NC 28608 USA

**Keywords:** Face masks, Race, Perceptions of trustworthiness, Threat, Anger

## Abstract

Despite their widespread use during the COVID-19 pandemic, face masks hinder abilities to interpret facial expressions. Yet, they can also reduce the appearance of characteristics that are used to categorize individuals into racial groups, such as Afrocentric features. The color of a face mask might also promote associations with certain types of behavior and professional occupations (e.g., blue surgical mask connoting physician stereotypes; black masks potentially being associated with criminality). This study assessed whether the presence and color of a face mask impacted perceptions of a target male of varying race. White participants (*N* = 250) were presented with an African American or White male adult face from the Chicago Face Database (of equal age and attractiveness) wearing a blue or black surgical mask, or no mask (Photoshopped onto the face) and rated the man on emotions (happy, sad, angry) as well as how trustworthy, threatening, and attractive the target appeared. Targets wearing a blue surgical mask were judged as more trustworthy and attractive than those wearing no mask (perhaps due to association with medical professions), but these judgements were not qualified by race, despite the African American target’s selection based on Afrocentric features. The color black on a face mask did not exacerbate negative perceptions of targets, perhaps suggesting a decline effect in previously demonstrated associations between this color and criminal actions. Unlike previous research performed at the beginning of the Covid-19 Pandemic on cloth masks shown to potentially exacerbate racial biases, surgical masks (pleated and made of polymeric materials), appear to lessen potential stereotyping of Black relative to White men.

## Introduction

In the winter of 2019, the world was made aware of SARS-CoV-2 (what many would call the COVID-19 virus), a respiratory virus that rapidly spread across the globe. By February 2022, there were over 383 million cases and 5.6 million deaths reported globally, with 74.7 million cases and 884 thousand deaths reported in the United States (World Health Organization [WHO] 2022). To help prevent the spread of the virus, many states mandated the use of face coverings in public areas (e.g., indoor spaces where social distancing could not occur, and public transportation). Many businesses provided masks if customers did not possess them upon entry.

The use of face masks sparked a country-wide debate concerning personal liberties and social responsibilities, polarizing America between mask advocates espousing the need to protect vulnerable populations (*I wear the mask for you*) and anti-maskers admonishing government oversight (Stewart 2020). Despite origins in health concerns and reduction in viral spread, mask usage during the COVID-19 Pandemic in early 2020 elucidated an issue with a longer and more pervasive history—racial disparity. Given the racialized stigmatization of clothing type for African American men, such as bandanas and hoodies (Gurung et al. 2020; Mason 2018), the pandemic seemed rife for exacerbating discrimination (Ruiz et al. 2020). Evidence that masks fueled concerns about negative stereotypes related to racial identity emerged, such that African American individuals showed higher rates of mask-induced police avoidance relative to other races studied (Kahn and Money 2021), including expressing concerns over being racially profiled for criminal activity if masked. Specific clothing items carrying “racialized” associations with gangs can act as a priming agent for African American stereotypes (Kahn and Money 2021; MacLin and Herrera 2006). This may help to explain why minorities wearing face masks during the early stages of the pandemic were more likely to perceive discrimination than those who did not, and this perceived discrimination was subsequently associated with increased mental distress (Liu et al. 2020).

Even in the absence of additional stigmatizing elements, racial stereotypes associated with African American men can be traced back in American history to the time of slavery and the Jim Crow era (Kleider-Offutt et al. 2017; Welch 2007). African American men are the most at risk for being a target of police brutality or even being killed by police in the U.S. (Edwards et al. 2019). Such stereotypes and negative biases regarding criminality and aggression tend to be particularly strong towards African American men with Afrocentric features—dark complexion, coarse hair, dark eye color, wide nose, and full lips (Hagiwara et al. 2012; Kleider-Offutt et al. 2017). Afrocentric features heighten racial associations with violence, aggression, and criminality (Kleider-Offutt et al. 2017), extend to other social contexts such as hiring decisions (e.g., Wade et al. 2004), and can trigger memory recognition errors due to such stereotype-consistent categorization (Kleider et al. 2012). The pervasiveness of these stereotypes is demonstrated by White individuals’ attentional reactivity to the faces of African American targets. For example, White participants performing a dot-probe task selectively attended to the faces of young African American men in the same way that individuals selectively attend to threats such as snakes, spiders, or angry faces (Trawalter et al. 2008).

*Masks and informational processing of faces*. There are conflicting perspectives on whether face masks would heighten racial stereotyping for African American targets. On the one hand, face masks may exacerbate racially-related threat perception by also reducing the ability to interpret emotions. When wearing a mask, the material tends to cover most of the cheek region (zygomaticus), limiting the capacity to observe and interpret positive facial expressions (Aue and Scherer 2008). Perceivers must then employ visual attention to the brow region of the face (corrugator), an area associated with a more negative valence expression. The potential for confusion in emotional recognition of faces was revealed by Cooper et al. (2022), who found that masked faces displaying emotions such as happiness or neutrality had more negative misattributions toward negative emotion than unmasked faces. Face coverings like a niqab (a veil worn by some Muslim women that covers most of the face except for the eyes) may also augment perceptions of negative emotions like anger or sadness, and accentuate negativity biases (Fischer et al. 2012). Likewise, happiness is more difficult to perceive when the lower part of the face is covered as the muscles involved in smiling are hidden from sight (Aue and Scherer 2008; Fischer et al. 2012).

Facial expressions can, concurrently, interact with racial categorization. For example, Hugenberg and Bodenhausen (2004) demonstrated that, likely because of the stereotypes associating African American individuals with hostility and aggression (Devine 1989), angry facial expressions elicited by a racially ambiguous target facilitated presumptions about race more so than happy expressions. If face masks reduce one’s ability to interpret the lower musculature facial feedback necessary to identify positive emotion, and individuals have existing trait attributions tendencies of heightened hostility regarding African American individuals, masks could augment racial stereotypes about the threat posed by an African American versus White person.

Despite reasons to believe that emotional recognition might be impaired by facial masks, fueling negative attributions related to race of a target, masks conceal the very characteristics that might quickly categorize racial identification. That is, characteristics that associate African American men with negative traits (e.g., large nose, full lips) could no longer be used when processing faces, thus, potentially reducing biases. Using normed data discriminating highest and lowest rated faces for trustworthiness, Oldmeadow and Koch (2021) used a sample of 40 African American and White faces from the Chicago Face Database and digitally altered them to wear a white mask. They demonstrated that irrespective of race, masks benefitted perceptions of trustworthiness, if in fact, the faces were judged as low in trustworthiness initially (Study 1), and increased perceptions of attractiveness across target races (Study 2). No evidence of racial profiling based on the presence of a mask was observed in this study. Similarly, Cooper et al. (2022) found no evidence of an own-race bias when performing emotional recognition of faces across masked or unmasked faces (i.e., recognition was poorer for masked faces irrespective of matching on race relative to unmasked faces). However, it is important to note that no behavioral attributions were assessed, only emotion accuracy measures.

In one of the first Pandemic-related investigations of race, mask wearing and trait related judgments, Christiani et al. (2022) found that face mask type and race did interact when making threat-related attributions about men. They presented participants with a White male or a African American male model across one of four conditions: no mask (surgical mask worn around the neck), surgical mask, homemade cloth mask, or a bandana (each of the masks/bandanas were light blue). They found that wearing a bandana or cloth mask led the African American target to be rated as more threatening and untrustworthy than when they were not wearing a mask, especially for respondents higher in racial resentment. However, the surgical mask did not increase perceived threateningness and untrustworthiness, a finding worth noting given that both mask types would be obstructing the ability to interpret facial expressions. This may have been due to an association between medical garb, including surgical masks, and perceptions of warmth and competence (Bogart et al 2003; Hareli et al. 2013) as well as trustworthiness and norms of social responsibility during the pandemic (Olivera-La Rosa et al. 2020). Interestingly, the bandana and cloth mask did not negatively influence perceptions of the White target, who was more favorably perceived when wearing a mask.

*Mask color and person judgment*. Based on reports from USA Today and NBC News, the color black was one of the most popular colors of face masks as the pandemic worsened (Campbell 2020; Malin 2020). This is interesting given some of the historical significance of the color black being linked cross-culturally to associations with evil and death (Adams and Osgood 1973) as well as fearful and sad emotions (Kaya and Epps 2004). In comparisons of black clothing to more neutral colors like gray, wearing black led to higher perceptions of aggression in confrontational situations (Vrji 1997). Linhartová et al. (2013) conducted a study examining the effect of black clothing in both men and women across contexts, including one of a more violent vs. more respectable nature. Regardless of context, a black-clothed man was rated as more aggressive than when wearing a lighter color, although the finding did not generalize to a woman wearing black.

Tham et al. (2020) argued for an interactional relationship between the psychological meaning of color of an object and the object itself. Whether colors evoke positively- or negatively-valenced emotional responses relates to both long-established associations with humans’ physical environments (e.g., black being associated with nighttime and darkness) and learned pairings between that color and an object or person through experience (Tham et al. 2020). For example, Tham et al. showed cross-culturally, consistent associations between symbolism and emotion with the color black Specifically, black was associated with negative concepts, including death and fear. What might these findings suggest for face masks? Given that the color black has associations with the induction of threat and fear, and certain masks exacerbate negative perceptions of Black men (Christiani et al. 2022), black face masks worn by Black men may have a reciprocal relationship. The coupling of preexisting threat-related stereotypes of African American men and the emotional ambiguity created by face coverings could lead to an even greater accentuation of perceived aggression and threateningness, particularly among White individuals. Gil and Le Bigot (2023) conducted one of the few studies examining mask color and recognition of emotion in facial expressions, using a cross-sectional comparison of children and young adults, who viewed faces expressing negative emotions (sadness, fear, and anger). Although they found that masks disrupted the recognition of sadness and fearfulness, contrary to expectations, they did not find evidence of a congruency effect between what they termed as *negatively-valenced* colors (red and black) compared to more positively associated colors (green and pink). Similarly, using a sample of university students, Blazhenkova et al. (2022) found that masks slowed processing, decreased confidence, as well as accuracy of emotion recognition, but this was more pronounced for black and patterned masks, relative to plain white masks.

It is unclear whether these results illuminate expectations about race and facial information, as the photographs were described as either being men or women, but racial identity was not indicated for targets. Whether mask color might yield different judgements for individuals of different races is unclear. For example, a blue cloth mask or bandana, when coupled with the preexisting stereotypes of African American men, did correlate with higher perceptions of threateningness and less trustworthiness, relative to a blue surgical mask, demonstrating that aspects of a mask, not just their presence, might interact with a perceiver’s racial categorization (Christiani et al. 2022). The color black on a surgical-type face mask may have higher associations with negative behavioral attributions more so than a blue surgical mask (traditionally associated with medical personnel), or no face mask. Additionally, the emotional ambiguity created by face coverings could lead to heightened perceived threateningness for African-American men relative to White men.

### Purpose of the present study

The purpose of the present study was to examine the influence of face mask color on judgements of traits (trustworthiness, attractiveness, and threateningness) and emotions (anger, happiness and sadness) of African American vs. White men, in a White sample of individuals. Christiani et al. (2022) argued that, “non-black respondents [would be] more likely than black respondents to be troubled by a black male wearing a mask” (p. 190), restricting their analyses to the former group. Thus, we elected to focus on a primarily White sample as well. Type of face mask (surgical texture, only varying in color) was held constant. We were particularly interested in whether the color black would hold negative connotations for judgments relative to the color blue (associated with medical settings). Secondarily, to our knowledge, this is the first study to select the African American target based on visible Afrocentric features. This allowed us to address whether a face mask led to a diminishing or exacerbation of negative judgement by concealing these features relative to when a White male target wore a face mask. If the color black triggers associations with negative behavior, despite a mask’s concealment of Afrocentric features, we might anticipate that a black mask worn by an African American man might lead to more activation of negative stereotypes, than the same mask on a White man.

A 2 (Race of Target: African American vs. White) X 3 (Mask Type: None, Blue surgical, vs. Black surgical) between-groups factorial study was utilized. Our key dependent measures were threateningness, trustworthiness, and perceptions of anger (based on previous inclusion by Christiani et al. (2022) for the target man. These were most related to attributions related to aggression and hostility (common stereotypes faced by African American men). We predicted a main effect for mask condition, such that regardless of race, black masks would increase ratings of threateningness and anger, and decrease ratings of trustworthiness. We also expected a main effect for race such that a negativity bias would show for the African American target for threateningness, anger, and trustworthiness for the African-American (selected because of Afrocentric features) relative to the White target.

Although the presence of a face mask has the potential to reduce the ability to categorize faces based on race, we predicted an interaction between face mask color and race (in line with Christiani et al. 2022), such that the black face mask would serve as a more salient cue for antisocial behavior when worn by an African-American target than a White target. That is, the negative associations between the color black, coupled with the coverage of the upper cheek region of the face to minimize the appearance of positive emotions would exacerbate racial stereotypes in White men. Specifically, the black mask was expected to elevate ratings of perceived threateningness and anger, but lower ratings of trustworthiness, for the African American man relative to the other mask color conditions. No differences were expected across levels of mask color for the White target.

There is contrasting evidence whether masks might reduce or enhance already existing stereotypic judgements surrounding race. On the one hand, they disrupt the processing of important emotional information. On the other, they serve to reduce the identification of facial features that lead to racial categorization (e.g., Afrocentric characteristics). Because of this, we used the following measures as exploratory dependent variables: happiness, sadness, and perceived attractiveness of the target. No predictions were made across mask condition for these dependent variables.

## Method

### Participants

This study was preregistered with OSF. A power analysis revealed that for a 95% confidence interval, *α* = 0.05, and an effect size of 0.32 (effect size observed in Oldmeadow & Koch 2021), 248 participants were required. We acquired 250 White participants (95 females, 154 males, one non-binary individual) via recruitment through Amazon Mechanical Turk (MTurk); *M*_age_ = 38.6 years old (*SD* = 10.5, range = 20–71). There was a relatively even distribution of individuals from various regions of the United States (24% from the Northeast, 27% from the Southeast, 18% from the Southwest, 22% from the Midwest, and 8% from the West). The majority of the sample held a bachelor’s degree or higher (68%). Participants were paid $0.75 as compensation.

### Materials

#### Photographs of masked targets

For the six experimental conditions (Race: African American or White; Mask: No Mask, Blue Surgical Mask, or Black Surgical Mask), photographs of a White male and a African American male were selected from the Chicago Face Database due to their similar ratings for age (*M* = 29) and attractiveness (*M* = 3.5; Ma et al. 2015). We tailored this procedure after Christiani et al.’s (2022), using one target for each racial category. Both photos showed a neutral facial expression, and the man wore a grey t-shirt against white background (see Appendix A). The picture of the African American man was originally chosen due to its use in the study by Kleider-Offutt et al. (2017) and was described as having stereotypical Afrocentric features. The photographs were then altered by a photo-editing software (Adobe Photoshop) to add an image of either a blue or black disposable surgical mask, which were acquired from online Amazon advertisements. For the current sample, ratings of the African American (*M* = 4.43, *SD* = 1.55) and White (*M* = 4.74, *SD* = 1.40) target photos yielded similar attractiveness ratings (*p* > 0.05). See Appendix A, Figures 3, 4, for photograph materials.

#### Traits and facial emotion items

Perceived trustworthiness, threateningness, and attractiveness were assessed on a 7-point bipolar scale (e.g., “Not Trustworthy” to “Very Trustworthy” on each side of the scale). Three items assessed happiness, sadness, and anger on a 7-point bipolar scale (“Not ___” to “Very ___”). Wording was as follows: “How trustworthy/threatening/attractive/etc., does the man in the photo seem to be?”.

## Procedure

Participants were given a link to an online Qualtrics survey and told the study was about the effect of face coverings on the perceptions of emotions in men and women. After affirming consent, participants were presented with a filler response target (an image of a biracial female wearing a pink cloth mask. This was done to reduce suspicion about racial bias being the focal point of the study) and were asked to rate her on the six different items regarding traits and emotions. Following this, participants were randomized to see one of the six picture conditions (i.e., we used a completely crossed experimental design) and were asked the same series of questions about the target traits and emotions. In total, participants were presented with two targets: the filler female target wearing a mask and the experimental target (African American or White man, in one of the three mask conditions). Demographic information regarding participants was completed before payment was delivered at the study’s conclusion.

## Results

### Test of main hypothesis

**Fig. 1:**
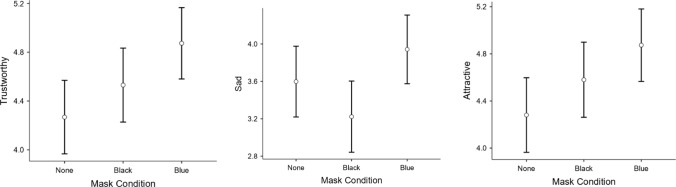
Estimated marginal means for ratings of trustworthiness, sadness, and attractiveness of targets as a function of mask color condition

Results for the 2 Race X 3 Mask condition factorial ANOVAs yielded no significant main effects or interactions for perceived threateningness (*F*s < 1.20, *p*s > 0.05, ɳ_p_^2^s < 0.010), or anger (all *F*s < 2.22, *p*s > 0.05, ɳ_p_^2^s < 0.010; see Table 1). However, for trustworthiness a significant main effect was found for the mask condition, *F*(2, 244) = 4.06, *p* = 0.018,* η*^*2*^_*p*_ = 0.032. Tukey’s post hoc analysis for the mask type demonstrated that the target with a blue surgical mask was rated by participants as more trustworthy than the target wearing no mask, 95% CI for mean difference [− 0.74, − 0.13] (see Table 1; Fig. 1). The target wearing the black mask was rated as no more trustworthy than either of the two targets. There was no main effect for race, however, (*F* < 1, *p* > 0.05). In other words, the African American target and White target were rated similarity across the dependent measure.^1^ The interaction also failed to reach significance.

**Table 1:** Overall and Marginal Means, *SD*s, *F*-values, *p*-values, and ɳ_p_^2^’s for Dependent Measures across Mask Type and Race of Target. Marginal Means that share a subscript are not statistically differently

	Blue mask Black Man White Man *M (SD) M (SD)*	Black mask Black Man White Man *M (SD) M (SD)*	No mask Black Man White Man *M (SD) M (SD)*	*F*	*p*	ɳ_p_^2^
Trustworthy	4.73 (1.34) 5.02 (1.32) Marginal *M*s: 4.87_a_ (1.33)	4.49 (1.54) 4.57 (1.23) 4.53_ab_ (1.38)	4.20 (1.27) 4.34 (1.62) 4.27_b_ (1.45)	4.06	**0.018**	0.032
Threateningness	3.50 (1.81) 3.74 (1.97) Marginal *M*s: 3.62_a_ (1.88)	3.46 (1.83) 3.17 (1.97) 3.31_a_ (1.90)	3.29 (1.54) 3.88 (1.87) 3.59_a_ (1.73)	0.69	0.503	0.006
Angry	3.89 (1.86) 3.63 (1.81) Marginal *M*s: 3.76_a_ (1.83)	3.69 (1.75) 3.05 (1.91) 3.36_a_ (1.85)	3.78 (1.71) 3.66 (1.82) 3.72_a_ (1.76)	1.14	0.321	0.009
	4.80 (1.34) 4.95 (1.19) Marginal *M*s: 4.87_a_ (1.26)	4.46 (1.59) 4.69 (1.55) 4.58_ab_ (1.56)	4.05 (1.41) 4.51 (1.63) 4.28_b_ (1.53)	3.51	**0.031**	0.028
Happy	3.05 (1.58) 4.16 (1.66) Marginal *M*s: 3.60_a_ (1.71)	3.10 (1.43) 3.98 (1.60) 3.56_a_ (1.57)	2.76 (1.48) 3.54 (1.95) 3.15_a_ (1.76)	1.93	0.148	0.016
Sad	3.61(1.99) 4.28 (1.69) Marginal *M*s: 3.94_a_ (1.87)	3.00 (1.65) 3.43 (1.78) 3.22_b_ (1.72)	3.10 (1.41) 4.10 (1.66) 3.60_ab_ (1.61)	**3.84**	**0.023**	**0.030**

Bolded *p*-values indicate significance below *α* = .05

### Exploratory analyses

Ratings of happiness and sadness were submitted to 2 × 3 ANOVAs. Main effects for race were found for both happiness (*F*[1, 244] = 20.11, *p* < 0.001, *η*^*2*^_*p*_ = 0.76) and sadness (*F*[1, 244] = 10.38, *p* = 0.001, *η*^*2*^_*p*_ = 0.04). On average, the White target (*M* = 3.99, *SD* = 1.74) was perceived as happier than the African American target (*M* = 3.12, *SD* = 1.63), 95% CI for mean difference [0.32, 0.82]. However, the White target (*M* = 3.94, *SD* = 1.74) was also perceived as sadder than the African American target (*M* = 3.25, *SD* = 1.72), 95% CI for mean difference [0.16, 0.66]. Note that neither CI for these emotional assessments contains 0.

As shown in Table 1, a main effect for mask condition emerged. Neither masked targets differed in perceived sadness from the non-masked target. However, the blue-masked target was perceived as sadder than the black-masked target (*p* = 0.02). No significant interactions occurred (for perceptions of happiness *F* < 0.24, *p* > 0.05; for sadness *F* < 0.58, *p* > 0.05; See Fig. 1.

**Fig. 2:**
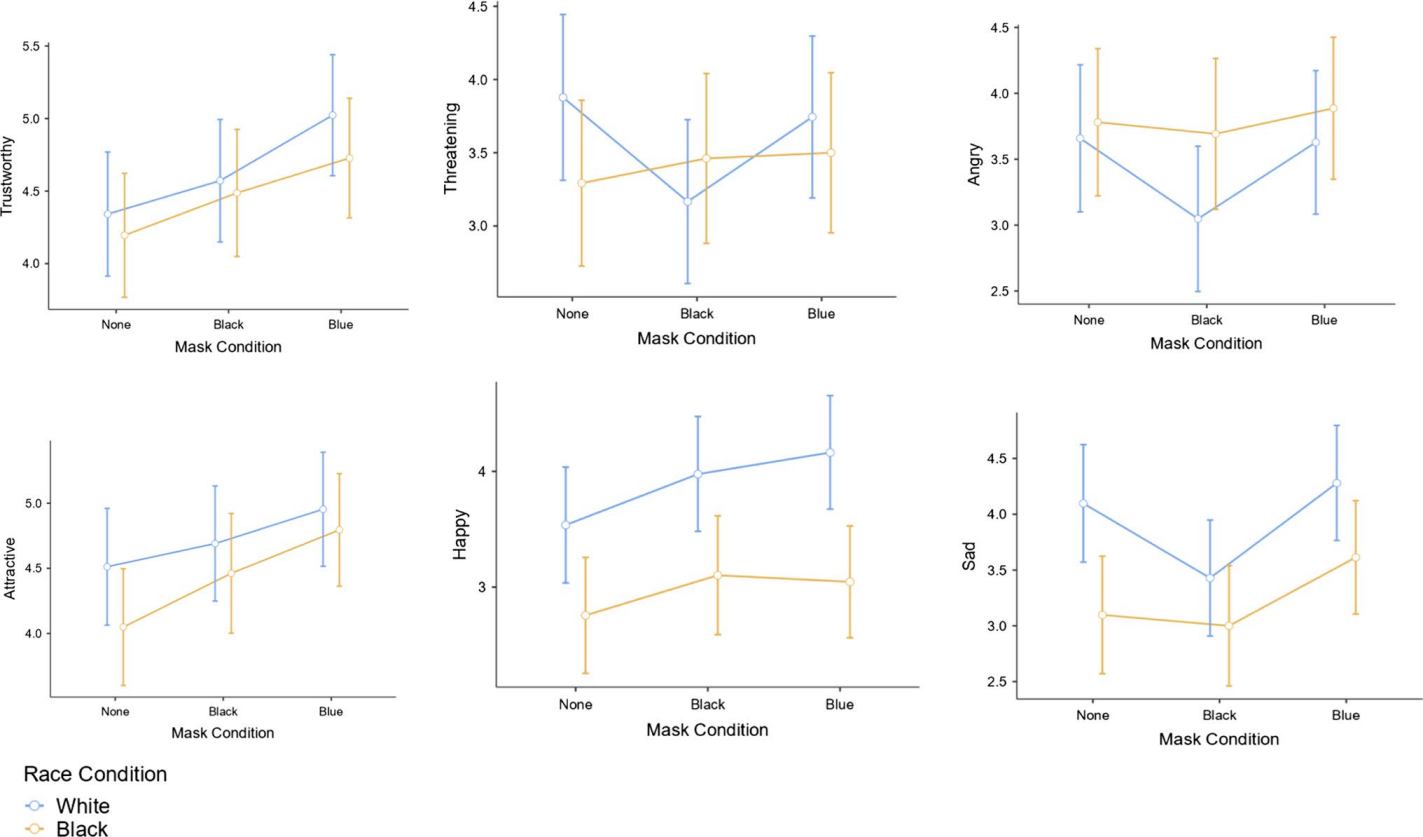
Estimated marginal means for ratings of trustworthiness, threateningness, anger, attractiveness, happiness, and sadness of targets as a function of mask color condition and race of target

**Fig. 3:**
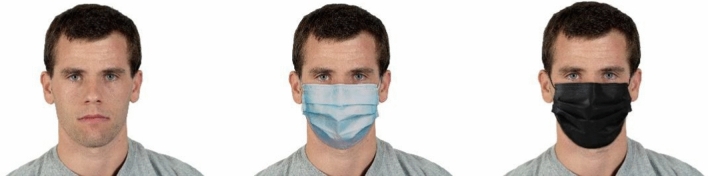
White male with no mask, blue mask, or black mask (Ref. number: CFD-WM-203-148N.jpg; Ma et al. 2015)

**Fig. 4:**
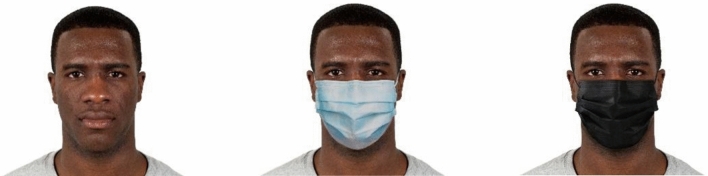
African American male with no mask, blue mask, or black mask. (Ref. number: CFD-BM-210-148N.jpg; Ma et al. 2015). Published with permission of Ma et al. (2015)

Finally, the 2 × 3 ANOVA for ratings of the target’s attractiveness demonstrated but one main effect for mask condition, *F*(2, 244) = 3.51, *p* = 0.031,* η*^*2*^_*p*_ = 0.028; See Table 1; Fig. 1. Tukey’s post-hoc comparisons showed that the blue surgical mask yielded higher attractiveness ratings for the target relative to the no mask condition, 95% CI for mean difference [− 0.71, − 0.13]. However, the black masked target did not differ from the others for attractiveness. No other significant effects emerged (neither the main effect for race, nor the interaction), all *F*s < 2.40, *p*s > 0.05, ɳ_p_^2^s < 0.020. Estimated marginal means for the fully crossed design are presented in Fig. 2.

## Discussion

Previous investigations following the COVID-19 Pandemic have been equivocal regarding whether face masks and their color exacerbate racial stereotypes. Using a sample of U.S. adult White men and women, this study found that blue surgical masks were associated with higher perceptions of trustworthiness and attractiveness of a male target relative to being unmasked. Trait judgments were not altered by the presence of a black mask, even when the target was an African American man with Afrocentric features. Christiani et al. (2022) demonstrated potentially negative influences of blue cloth face masks/bandanas for an African American man on perceptions of trustworthiness and threateningness, but not a White man. In previous research, blue surgical masks did not afford more negative perceptions, regardless of race (Christiani et al. 2022) nor did a plain white cloth mask (Oldmeadow and Koch 2021), which was found to enhance social judgement for both African American and White men.

Our results corroborate findings that blue surgical masks may afford positive associations to a wearer (Bogart et al 2003; Hareli et al. 2013; Olivera-La Rosa et al. 2020). That is, there was no support for our hypothesis that face masks would exacerbate negative perceptions of African American men relative to White men, especially if the mask was black in color. The black surgical mask did not influence negative perceptions of either target, countering previous research associating the color black with more perceived aggression in men (Vrij 1997; Linhartová et al. 2013).

It appears that our standardization of mask type (blue and black had the textured appearance of a surgical mask, was pleated, and with ear loops) rendered the black mask to be characteristically similar to the more traditional surgical mask. Despite this, the more traditional blue mask led perceivers to judge the target as more trustworthy *and* more attractive than the non-masked individual, but not less threatening or angry. Heuristically, it may have heightened accessibility of norms surrounding responsibility and trust connected to physicians (Olivera-La Rosa et al. 2020). Black surgical masks seem to be a more novel phenomenon relative to blue or white masks previously associated with healthcare settings, evolving from the Covid pandemic. The black mask that we used shared features of blue surgical masks and black cloth masks—a hybrid of sorts.

Black masks have also been shown to be a preferred color among mask wearers (Blazhenkova et al. 2022), so their popularity may contribute to a dilution in negative attributions made when others wear them, regardless of race. Based on principles of attraction, mere exposure to a novel stimulus can reduce uncertainty and create a greater preference toward that object (Lee 2001). Thus, exposure to a stranger wearing a familiar colored masked might reduce apprehensions one might normally have toward that person. Further, if color is “context-sensitive”, holding varied associations across settings (e.g., the color red’s associations with love but also with danger) (Tham et al. 2020), the presentation of either color (black or blue) in the form of a surgical mask may have provided a salient context (cognitive connections to medical professionals) that superseded color distinctions shown to occur when other associations are made relevant. Thus, surgical masks provide such a clear context connection, that the color black’s associations with more negative meaning (e.g., fear) is inhibited.

Despite previous discussions by Kleider-Offutt et al. (2017) indicating that men with more Afrocentric features tend to be associated with increased perceptions of threateningness, aggression, and violence, our target exhibiting those features was not rated more negatively than the White target. This may be due to a heightened awareness of discrimination towards African American individuals as a result of the *Black Lives Matter* movement during the summer of 2020. Also, masks likely conceal features that help in identifying races—much like Oldmeadow and Koch’s (2021) findings that masks led to more positive perceptions of faces originally judged as “less” trustworthy possibly because trustworthiness judgements are impacted by the degree to which people can see the nose and mouth regions of a face (Santos and Young 2011). Similarly, attractiveness judgements are elevated when eye regions are more prominent (Santos and Young 2011).

Fischer et al. (2012) found that face coverings reduce the ability to read emotions and may also exacerbate racial stereotypes. We did not find that face mask color differentially impacted judgements of emotion for men of varying race, but we did find that that the African American man in this study was rated as less sad and less happy than the White target. Since we intentionally selected target photographs of similar attractiveness and age, it may be that our participants perceived that emotional expressions were more difficult to detect for our African American target compared to our White target. This was not the case, however, for anger. Although we did not measure attentional allocation to targets, Friesen et al. (2019) found that White participants spent less time attending to the eyes of White than African American faces (as measured by eye tracking technology), which subsequently predicted accuracy in interpreting happiness in those faces.

Additionally, our White sample may have made more assumptions about reading emotions of a same-race compared to an other-race target, or an own-race bias for emotional recognition (Elfenbein and Ambady 2003). In other words, the interpretation of the African American target’s happiness and sadness were more muted than those of the White target. These findings align with Friesen et al.’s showing that White participants showed better accuracy when discriminating true versus false smiles on the faces of White as compared to African American targets. Despite positive strides toward less racially related stereotypes involving perceived hostility in the form of anger for White individuals in our study, these findings support continued bias for how Black facial emotions are processed relative to White faces.

Of course, it is important to note that group identification can extend well beyond racial categorization. As stated earlier, the Covid Pandemic created tensions over the wearing of masks, dichotomizing people into natural in-groups and out-groups. Such potential for in-group favoritism has been shown for both mask and non-mask wearers in prisoner’s dilemma simulations (Powdthavee et al. 2021). Social identification with mask wearing may draw more attention when processing faces as an own-group bias than racial categorization (e.g., see Van Bavel and Cunningham 2012).

## Limitations

It would be beneficial to replicate this research with a more diverse sample of participants. Our MTurk sample included a wide range of ages, but an overrepresentation of participants with at least a bachelor’s level education, warranting some concerns regarding the limitations of WEIRD samples (Henrich et al. 2010). Some research has found that MTurk samples lean more ideologically liberal (Berinsky et al. 2012), potentially attenuating our findings for racial stereotyping. Diversifying the racial targets to include other minority groups and genders is also important. Since stereotypic perceptions of threat are so relevant to African American men as targets, we focused on stimulus photos of men, but realize that this restricts the ability to generalize to women.

The sample comprised a larger percentage of White male participants (62%) relative to female participants. This is relevant because mask-wearing at the height of the Covid Pandemic showed disparities in wearing masks to reduce the spread of Covid along racial and gendered lines (Hearne and Niño, 2022). For example, one cross-sectional, nationally representative household survey found that among White, Latina, Asian, and Black demographic groups, White men were the least likely to engage in wearing a mask. Furthermore, such behavioral choices correspond with heightened perceptions of toughness and masculinity (Palmer and Peterson 2020). We did not ask participants if they, themselves, wore masks during the pandemic, so it is unclear whether participants’ own behavioral practices, particularly those of the men in our sample, created biases against either of our masked targets.

Furthermore, our design was limited to two targets (controlling for age and attractiveness as reported by Ma et al. 2015), who varied by both perceived race and Afrocentric. This restricted the ability to determine whether various aspects of Afrocentricity in faces, not covered by a mask (e.g., hair texture), might alter outcomes. For example, Blair et al. (2004) found in a random sample of inmate records in the state Florida that White and African American defendants with roughly equivalent criminal histories did not receive harsher judge-issued prison sentences. Importantly, comparisons of sentences within racial groups revealed that men with more Afrocentric features did receive harsher sentences than those with less racially stereotypical features. Utilization of a within-groups design, including adding more stimulus faces to our design, will enable a better determination of how variations in skin tone, hair texture, etc., affect perceptions of racial groups when a face is masked.

There are additional considerations regarding the nature of color perception that warrant discussion. Spence (2015) supported the bottom-up processing of color sensory cues related to taste and how color creates expectancies surrounding flavor. The intensity of color, for example, can create the presumption, that a food will have a similarly robust flavor. It is possible that the particular shades of face mask colors that we used created particular expectancies about our targets that affected trait and behavioral judgments. The current study was limited by the selection of only one hue for either black or blue, with no variations of lightness or darkness, elements that can attenuate or increase emotional and meaning-related associations with color (Tham et al. 2020). Participants were given little context in these evaluations, being only told that the study was about “face masks and emotional perceptions,” so the complexity of color associations was restricted.

Future research could also investigate how the color of different types of face masks, such as a black cloth mask or bandana, could influence perceptions of individuals. Bandanas’ associated connection with racial stigma and urban “swagger” (Gurung et al. 2020; Mason 2018) would likely heighten activation of stereotypes connected with gang membership and criminality (MacLin and Herrera 2006). This could be particularly true given Tham et al.’s (2020) discussion of reciprocity between the physical color of an object and previous associations between an object possessing that color and learned experience.

Finally, the static nature of judging photographs undermines the realities and complexities of racial prejudice and discrimination. Face-to-face encounters are more likely to reveal inequities and the subtle forms of prejudice commonly faced by minorities. These “microaggressions” occur in brief exchanges, where messages of disparagement are conveyed in understated ways either verbally or nonverbally (Solórzano et al. 2000; Sue et al. 2007). Such behaviors likely contribute to minority group’s perceived inequitable treatment during the pandemic when wearing masks (Kahn and Money 2021). More authentic examinations of how masks might influence individual’s health-practices are warranted, not only for the recent Pandemic, but for future events moving forward.

## Conclusion

The juncture of a global pandemic still encouraging mask wearing to reduce illness spread, and enduring resentment toward African Americans decries the continued investigation of factors that attenuate prejudice. Governmental policy that is directed toward public health must consider whether particular groups will be disadvantaged or more negatively impacted than others when such strategies are implemented. Our findings align with Christiani et al.’s (2022) recommendation that measures be taken to ensure equal access to beneficial and protective measures during health crises (e.g., surgical masks being made widely available, especially in African American communities, with concurrent educational messaging about the health benefits of surgical masks to decrease disease spread). The association of traditionally colored surgical face masks with prosocial behaviors in medical settings, although potentially minimizing emotional detection in other-race faces, may serve to buffer negative evaluations surrounding racial stereotyping of African American faces. Black-colored surgical masks do not appear to carry social liability for judgement relative to blue masks. This is encouraging such that masks have been shown to help minimize the spread of SARS-CoV-2 (e.g., see Boulos et al. 2023), and superficial aspects of surgical masks could serve as an incentive for wearing masks (e.g., color choices) without prompting negative judgments for those who wear them.

## Data Availability

Data are available and housed in OSF’s data repository under the corresponding author’s name at:

## Appendix A

Photographs of masked targets see Figs. 3, 4.

## Footnotes

1 The 4-item Racial Resentment Scale (Wilson & Davis; 2011) was included as a means of assessing participants’ level of unsympathetic-ness toward African Americans. A median split of the scale was used as a categorical variable (low vs. high) and included as a factor in the analyses. No significant interaction emerged between level of racial resentment and race of target for perceived threateningness, trustworthiness, or anger (all *F*s < 2, *p*s > 0.05).

## References

[CR1] Adams FM, Osgood CE (1973) A cross-cultural study of the affective meanings of color. J Cross Cult Psychol 4(2):135–156

[CR2] Aue T, Scherer KR (2008) Appraisal-driven somatovisceral response patterning: effects of intrinsic pleasantness and goal conduciveness. Biol Psychol 79(2):158–164. 10.1016/j.biopsycho.2008.04.004 18495321 10.1016/j.biopsycho.2008.04.004

[CR3] Berinsky A, Huber G, Lenz G (2012) Evaluating online labor markets for experimental research: amazon.com’s mechanical Turk. Political Anal 20(3):351–368

[CR4] Blair IV, Judd CM, Chapleau KM (2004) The influence of Afrocentric facial features in criminal sentencing. Psychol Sci 15(10):674–679. 10.1111/j.0956-7976.2004.00739.x 15447638 10.1111/j.0956-7976.2004.00739.x

[CR5] Blazhenkova O, Dogerlioglu-Demir K, Booth RW (2022) Masked emotions: do face mask patterns and colors affect the recognition of emotions? Cogn Res 7:33. 10.1186/s41235-022-00380-y 10.1186/s41235-022-00380-y PMC8990494 35394218

[CR6] Bogart LM, Bird ST, Walt LC, Delahanty DL, Figler JL (2003) Association of stereotypes about physicians to health care satisfaction, help-seeking behavior, and adherence to treatment. Soc Sci Med 58:1049–1058. 10.1016/S0277-9536(03)00277-6 10.1016/s0277-9536(03)00277-6 14723901

[CR7] Boulos L, Curran JA, Gallant A, Wong H, Johnson C, Delahunty-Pike A, Saxinger L, Chu D, Comeau J, Flynn T, Clegg J, Dye C (2023) Effectiveness of face masks for reducing transmission of SARS-CoV-2: a rapid systematic review. Philos Trans Ser A Math Phys Eng Sci 381(2257):20230133. 10.1098/rsta.2023.0133 37611625 10.1098/rsta.2023.0133 PMC10446908

[CR8] Campbell C (2020) 10 popular face masks everyone is buying on Amazon. https://www.usatoday.com/story/tech/reviewedcom/2020/07/21/10-most-popular-facemasks-you-can-buy-amazon/5482759002/

[CR9] Christiani L, Clark C, Greene S, Hetherington M, Wager E (2022) Masks and racial stereotypes in a pandemic: the case for surgical masks. J Race Ethn Polit 7(2):185–202. 10.1017/rep.2021.9

[CR10] Cooper H, Brar A, Beyaztas H, Jennings B, Bennetts R (2022) The effects of face coverings, own-ethnicity biases, and attitudes on emotion recognition. Cogn Res 7:57. 10.1186/s41235-022-00400-x 10.1186/s41235-022-00400-x PMC9250564 35780221

[CR11] Devine PG (1989) Stereotypes and prejudice: their automatic and controlled components. J Pers Soc Psychol 56(1):5. 10.1037/0022-3514.56.1.5

[CR12] Edwards F, Lee H, Esposito M (2019) Risk of being killed by police use of force in the United States by age, race–ethnicity, and sex. Proc Natl Acad Sci 116(34):16793–16798. 10.1073/pnas.1821204116 31383756 10.1073/pnas.1821204116 PMC6708348

[CR13] Elfenbein HA, Ambady N (2003) When familiarity breeds accuracy: cultural exposure and facial emotion recognition. J Pers Soc Psychol 85(2):276–290. 10.1037/0022-3514.85.2.276 12916570 10.1037/0022-3514.85.2.276

[CR14] Fischer AH, Gillebaart M, Rotteveel M, Becker D, Vliek M (2012) Veiled emotions: the effect of covered faces on emotion perception and attitudes. Soc Psychol Personal Sci 3(3):266–273. 10.1177/1948550611418534

[CR15] Friesen JP, Kawakami K, Vingilis-Jaremko L, Caprara R, Sidhu DM, Williams A, Hugenberg K, Rodríguez-Bailón R, Cañadas E, Niedenthal P (2019) Perceiving happiness in an intergroup context: the role of race and attention to the eyes in differentiating between true and false smiles. J Pers Soc Psychol 116(3):375–395. 10.1037/pspa0000139 30614725 10.1037/pspa0000139

[CR16] Gil S, Le Bigot L (2023) Emotional face recognition when a colored mask is worn: a cross-sectional study. Sci Rep 13(1):174. 10.1038/s41598-022-27049-2 36599964 10.1038/s41598-022-27049-2 PMC9812539

[CR17] Gurung RAR, Stoa R, Livingston N, Mather H (2020) Can success deflect racism? Clothing and perceptions of African American men. J Soc Psychol 161(1):119–128. 10.1080/00224545.2020.1787938 32597345 10.1080/00224545.2020.1787938

[CR18] Hagiwara N, Kashy DA, Cesario J (2012) The independent effects of skin tone and facialfeatures on Whites’ affective reactions to Blacks. J Exp Soc Psychol 48:892–898. 10.1016/j.jesp.2012.02.001

[CR19] Hareli S, David S, Hess U (2013) Competent and warm but unemotional: the influence of occupational stereotypes on the attribution of emotions. J Nonverbal Behav 37:307–317. 10.1007/s10919-013-0157-x

[CR20] Hearne BN, Niño MD (2022) Understanding how race, ethnicity, and gender shape mask-wearing adherence during the COVID-19 pandemic: evidence from the COVID impact survey. J Racial Ethn Health Dispar 9(1):176–183. 10.1007/s40615-020-00941-1 10.1007/s40615-020-00941-1 PMC7814861 33469866

[CR21] Henrich J, Heine SJ, Norenzayan A (2010) The weirdest people in the world? Behav Brain Sci 33:61–83. 10.1017/S0140525X0999152X 20550733 10.1017/S0140525X0999152X

[CR23] Hugenberg K, Bodenhausen GV (2004) Category membership moderates the inhibition of social identities. J Exp Soc Psychol 40(2):233–238. 10.1111/j.0956-7976.2004.00680.x

[CR24] Kahn KB, Money EEL (2021) (Un)masking threat: racial minorities experience race-based social identity threat wearing face masks during COVID-19. Group Process Intergroup Relat 25(4):871–891. 10.1177/1368430221998781

[CR25] Kaya N, Epps HH (2004) Relationship between color and emotion: a study of college students. Coll Stud J 38(3):396–405

[CR26] Kleider HM, Cavrak SE, Knuycky LR (2012) Looking like a criminal: stereotypical black facial features promote face source memory error. Mem Cognit 40:1200–1213. 10.3758/s13421-012-0229-x 22773417 10.3758/s13421-012-0229-x

[CR27] Kleider-Offutt HM, Bond AD, Hegerty SE (2017) Black stereotypical features: when a face type can get you in trouble. Curr Dir Psychol Sci 26(1):28–33. 10.1177/0963721416667916

[CR28] Lee AY (2001) The mere exposure effect: an uncertainty reduction explanation revisited. Pers Soc Psychol Bull 27(10):1255–1266. 10.1177/01461672012710002

[CR29] Linhartová P, Tapal A, Brabenec L, Macecek R, Buchta JJ, Procházka J, Ježek S, Vaculík M (2013) The color black and situational context: factors influencing perception of an individual’s aggressiveness and respectability. Studia Psychol 55(4):321–333. 10.21909/sp.2013.04.646

[CR30] Liu Y, Finch BK, Brenneke SG, Thomas K, Le PD (2020) Perceived discrimination and mental distress amid the COVID-19 pandemic: evidence from the understanding America study. Am J Prev Med 59(4):481–492. 10.1016/j.amepre.2020.06.007 32829968 10.1016/j.amepre.2020.06.007 PMC7336127

[CR31] Ma DS, Correll J, Wittenbrink B (2015) The chicago face database: a free stimulus set of faces and norming data. Behav Res Methods 47(4):1122–1135. 10.3758/s13428-014-0532-5 25582810 10.3758/s13428-014-0532-5

[CR32] MacLin MK, Herrera V (2006) The criminal stereotype. North Am J Psychol 8:197–208

[CR33] Malin Z (2020) Face masks: The most purchased masks we’ve recently covered. NBC News. https://www.nbcnews.com/shopping/apparel/popular-face-masks-cdc-valvesn1236806

[CR34] Mason W (2018) ‘Swagger’: urban youth culture, consumption and social positioning. Sociology 52(6):1117–1133. 10.1177/0038038517698638

[CR35] Oldmeadow JA, Koch C (2021) Effects of Face Masks on Person Perception. Perception 50(10):876–889. 10.1177/03010066211045172 34549649 10.1177/03010066211045172

[CR36] Olivera-La Rosa A, Chuquichambi EG, Ingram G (2020) Keep your (social) distance: pathogen concerns and social perception in the time of COVID-19. Personal Individ Differ 166:110200. 10.1016/j.paid.2020.110200 10.1016/j.paid.2020.110200 PMC7296322 32834278

[CR37] Palmer CL, Peterson RD (2020) Toxic mask-ulinity: the link between masculine toughness and affective reactions to mask wearing in the COVID-19 Era. Politics Gend. 10.1017/S1743923X20000422

[CR38] Powdthavee N, Riyanto YE, Wong ECL, Yeo JXW, Chan QY (2021) When face masks signal social identity: explaining the deep face-mask divide during the COVID-19 pandemic. PLoS ONE 16(6):e0253195. 10.1371/journal.pone.0253195 34111233 10.1371/journal.pone.0253195 PMC8191909

[CR39] Ruiz NG, Horowitz JM, Tamir C (2020) Many black and Asian Americans say they have experienced discrimination amid the COVID-19 outbreak. Pew Research Center. https://www.pewresearch.org/social-trends/2020/07/01/many-black-and-asian-americans-say-they-have-experienced-discrimination-amid-the-covid-19-outbreak/

[CR40] Santos IM, Young AW (2011) Inferring social attributes from different face regions: evidence for holistic processing. Q J Exp Psychol 64(4):751–766. 10.1080/17470218.2010.519779 10.1080/17470218.2010.519779 21086218

[CR41] Solórzano D, Ceja M, Yosso T (2000) Critical race theory, racial microaggressions, and campus racial climate: the experiences of African American college students. J Negro Educ 69:60–73

[CR42] Spence C (2015) On the psychological impact of food colour. Flavour 4(21):1–16. 10.1186/s13411-015-0031-3

[CR43] Stewart E (2020) Anti-maskers explain themselves. Vox. https://www.vox.com/the-goods/2020/8/7/21357400/anti-mask-protest-rallies-donald-trump-covid-19

[CR44] Sue DW, Capodilupo CM, Torino GC, Bucceri JM, Holder AMB, Nadal KL, Esquilin M (2007) Racial microaggressions in everyday life: implications for clinical practice. Am Psychol 62(4):271–286. 10.1037/0003-066X.62.4.271 17516773 10.1037/0003-066X.62.4.271

[CR45] Tham DSY, Sowden PT, Grandison A, Franklin A, Lee AKW, Ng M, Park J, Pang W, Zhao J (2020) A systematic investigation of conceptual color associations. J Exp Psychol Gen 149(7):1311–1332. 10.1037/xge0000703 31763867 10.1037/xge0000703

[CR46] Trawalter S, Todd AR, Baird AA, Richeson JA (2008) Attending to threat: race-based patterns of selective attention. J Exp Soc Psychol 44:1322–1327. 10.1016/j.jesp.2008.03.006 19727428 10.1016/j.jesp.2008.03.006 PMC2633407

[CR47] Van Bavel JJ, Cunningham WA (2012) A social identity approach to person memory: group membership, collective identification, and social role shape attention and memory. Personal Soc Psychol Bull 38(12):1566–1578. 10.1177/0146167212455829 10.1177/0146167212455829 22914991

[CR48] Vrij A (1997) Wearing black clothes: the impact of offenders’ and suspects’ clothing on impression formation. Appl Cogn Psychol 11(1):47–53

[CR49] Wade TJ, Romano MJ, Blue L (2004) The effect of African American skin color on hiring preferences. J Appl Soc Psychol 34(12):2550–2558. 10.1111/j.1559-1816.2004.tb01991.x

[CR50] Welch K (2007) Black criminal stereotypes and racial profiling. J Contemp Crim Justice 23(3):276–288. 10.1177/1043986207306870

[CR51] Wilson DC, Davis DW (2011) Reexamining racial resentment: conceptualization and content. Ann Am Acad Pol Soc Sci 634(1):117–133. 10.1177/0002716210388477

[CR52] World Health Organization (2022) WHO Coronavirus (COVID-19) Dashboard. World Health Organization. https://covid19.who.int/

